# A self-powered ingestible wireless biosensing system for real-time in situ monitoring of gastrointestinal tract metabolites

**DOI:** 10.1038/s41467-022-35074-y

**Published:** 2022-12-01

**Authors:** Ernesto De la Paz, Nikhil Harsha Maganti, Alexander Trifonov, Itthipon Jeerapan, Kuldeep Mahato, Lu Yin, Thitaporn Sonsa-ard, Nicolas Ma, Won Jung, Ryan Burns, Amir Zarrinpar, Joseph Wang, Patrick P. Mercier

**Affiliations:** 1 Department of Nanoengineering, University of California San Diego, La Jolla, CA 92093 USA; 2 Department of Electrical and Computer Engineering, University of California San Diego, La Jolla, CA 92093 USA; 3 Division of Gastroenterology, University of California San Diego, La Jolla, CA 92093 USA; 4 VA San Diego Healthcare System, La Jolla, CA 92161 USA; 5 Center for Microbiome Innovation, University of California San Diego, La Jolla, CA 92093 USA

**Keywords:** Biomedical engineering, Fuel cells, Diagnostic markers, Biosensors

## Abstract

Information related to the diverse and dynamic metabolite composition of the small intestine is crucial for the diagnosis and treatment of various diseases. However, our current understanding of the physiochemical dynamics of metabolic processes within the small intestine is limited due to the lack of in situ access to the intestinal environment. Here, we report a demonstration of a battery-free ingestible biosensing system for monitoring metabolites in the small intestine. As a proof of concept, we monitor the intestinal glucose dynamics on a porcine model. Battery-free operation is achieved through a self-powered glucose biofuel cell/biosensor integrated into a circuit that performs energy harvesting, biosensing, and wireless telemetry via a power-to-frequency conversion scheme using magnetic human body communication. Such long-term biochemical analysis could potentially provide critical information regarding the complex and dynamic small intestine metabolic profiles.

## Abstract (web summary)

Metabolic dynamics within the small intestine are difficult to study due to the lack of in situ access. Here, the authors report an ingestible, self-powered, and wireless biosensing system, demonstrating proof-of-principle real-time glucose monitoring in the small intestines of pigs.

## Introduction

Approximately one in five people suffer from gastrointestinal (GI) disorders at some stage of their lives, with these diseases accounting for considerable health care utilization and costs^1–3^. As a result, gut microbiota-derived metabolites and their role in diseases, nutrition, obesity, and other areas have attracted significant attention^4–7^. Research studies have suggested that some metabolites in the intestinal region of the GI tract play a crucial role in gut functioning. For instance, chronic diseases such as inflammatory bowel disease (IBD), diabetes, or obesity are caused by the dysfunction of the intestinal processes involving the absorption or digestion of metabolites in the gut. Therefore, access to relevant sections of the digestive tract, such as the small intestine, could facilitate the diagnosis and treatment of many GI disorders^8–10^. Regardless of the significant breakthroughs achieved in the diagnosis of functional aspects of the gut microbiome, monitoring of metabolites in the distal area of the stomach is still performed using invasive procedures or non-real-time analysis such as stool tests^11^ endoscopic fluid collection^12^ or breath testing^13^.

Miniaturized ingestible device modules offer a great promise in accessing the gut microbiota^14^. In fact, swallowable, pill-sized capsules have recently emerged as a way to interrogate various other operations in the GI environment, including imaging and endoscopy^15,16^_,_ core temperature^17–19^_,_ heart rate^20^, pH^21,22^, pressure^23–25^, hemorrhage^26^, and monitoring of medication^27,28^. The application of ingestible devices has been further expanded to monitoring biomarkers of interest in different sections of the digestive tract. For instance, biomarkers found during acute bleeding events in the stomach were detected by luminescence readouts with the assistance of a bacterial-based biosensor integrated into an ingestible device^29^. Also, capsule-based gas sensing devices have been used for the detection of inflammatory biomarkers in the intestine^30^ and gases produced along the gut by dietary alteration in human subjects^31^.

Despite the tremendous progress in the field of ingestible devices, and more specifically those targeting the intestinal environment, no solutions exist today to capture the dynamic profile of metabolites in this region in real-time. In addition, most prior works related to ingestibles make use of batteries. This is in part due to the high-power demands of wireless communications, as fundamental trade-offs between antenna size and far-field radiation efficiency governed by the Wheeler–Chu limit^32,33^ and absorption of electromagnetic power in tissue add to significant losses. Also, batteries contain toxic elements that in direct exposure to the gut environment can potentially result in serious complications^34^. These reasons may restrict the long-term monitoring processes and even lead to severe complications in the absence of potentially bulky hermetic packaging^35,36^. Efforts to eliminate the need for batteries have focused on developing the next generation of self-powered battery-free systems. Their application for simultaneous monitoring of biomarkers and energy harvesting has been illustrated, however, such applications have not demonstrated real-time metabolite monitoring^37–39^ and instead focused on other parameters such as physiological temperature changes inside a porcine model^40^.

Herein, we present a self-powered, battery-free, wireless, energy-efficient biosensing capsule for monitoring glucose dynamics in the small intestine. This system integrates multiple features toward addressing key challenges for realizing capsule-type ingestible in situ chemical sensing devices. The capsule incorporates a glucose biofuel cell (BFC) for obtaining power during operation, while simultaneously measuring changing glucose concentrations from the extracted power itself. We employ an energy-efficient magnetic human body communication (mHBC) scheme operating in the 40–200 MHz range to receive the time-resolved transmitted signals. The incorporation of mHBC has been shown to efficiently reduce the electromagnetic energy losses towards the microwatt-level in wearable applications^41,42^, which is the level needed to enable operation from a small BFC-powered capsule located inside the GI tract.

**Fig. 1:**
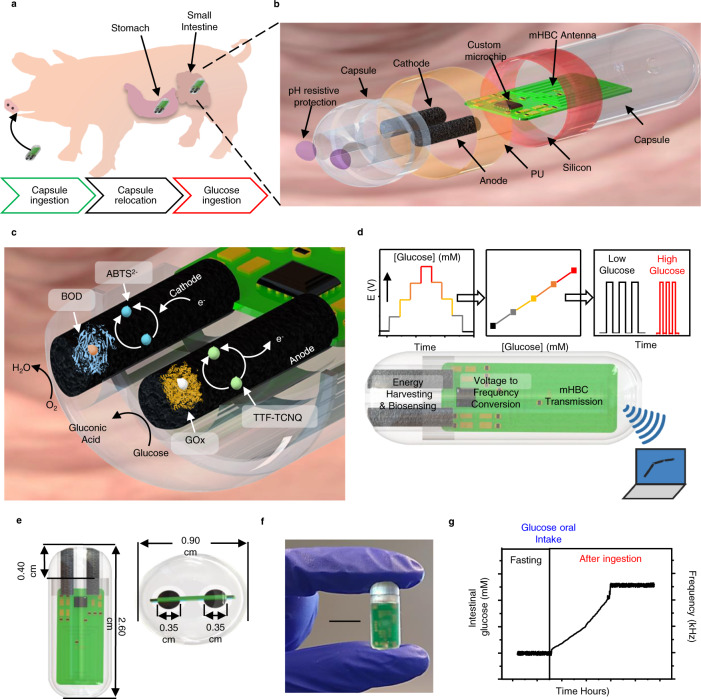
Design and sensing mechanism of the ingestible self-powered BFC capsule sensor. **a** Schematics of the capsule sensor operating in a porcine model. **b** Schematic layout of the capsule sensor. **c** Illustration of the chemical composition and reaction mechanisms inside the BFC sensor. **d** Signal conversion mechanism converting glucose concentrations to voltage, then to mHBC modulation signal frequency, transmitted wirelessly to an external receiver. **e** Dimensions of the capsule (length: 2.6 cm, diameter: 0.9 cm) and of the internal BFC electrodes (sensing length: 0.40 cm, diameter: 0.35 cm). **f** Picture of the actual device. Scale bar, 1 cm. **g** Example of measured data from in situ experiments in a porcine model upon oral glucose intake.

This pilot study uses a porcine model due to its anatomical and physiological similarities to the human GI tract^43^. The device is delivered orally and safely passes through the highly acidic stomach media before reaching the small intestine, where continuous glucose sensing occurs (Fig. 1a). The physical design, shown in Fig. 1b, indicates the construction of the capsule. A 3D-printed shell is used to compactly encapsulate the biosensing components, including BFC electrodes, an integrated circuit, and a mHBC antenna. A pH-responsive enteric coating is adapted for temporary protection of the BFC from the acidic stomach environment, which thereafter dissolves in the pH-neutral intestinal medium, while the silicone/polyurethane (PU) coatings ensured the insulation of the electronics. Figure 1c displays the composition and reaction mechanisms of the BFC. Both the anode and cathode are constructed on carbon nanotube (CNT) coated Ni-foam. The anode performs a glucose oxidation reaction using glucose oxidase (GOx), assisted by the tetrathiafulvalene-7,7,8,8-tetracyanoquinodiamethane (TTF-TCNQ) mediator, while the cathode performs an oxygen reduction reaction employing bilirubin oxidase (BOD) and 2,2′-azino-bis(3-ethylbenzothiazoline-6-sulfonic acid (ABTS^2−^) as a mediator. The BFC’s voltage under a given load is correlated with the concentration of glucose upon calibration, and then converted to a frequency signal and transmitted using the custom integrated circuit (Fig. 1d). The self-powered battery-free operation and low energy-consumption design conserve precious volume to enable significant system miniaturization (*l* = 2.6 cm, ⍉ = 0.9 cm) along with the full integration of sensing, digitization, wireless communication, and energy harvesting functions, while ensuring its efficient operation (Fig. 1e, f). Figure 1g exemplifies a typical output from the self-powered ingestible biosensor during in situ operation, recorded upon providing glucose-containing solutions to the animal, demonstrating a positive shift in the frequency signal upon elevation of glucose levels.

## Results

### Electronic circuit design and characterization

**Fig. 2:**
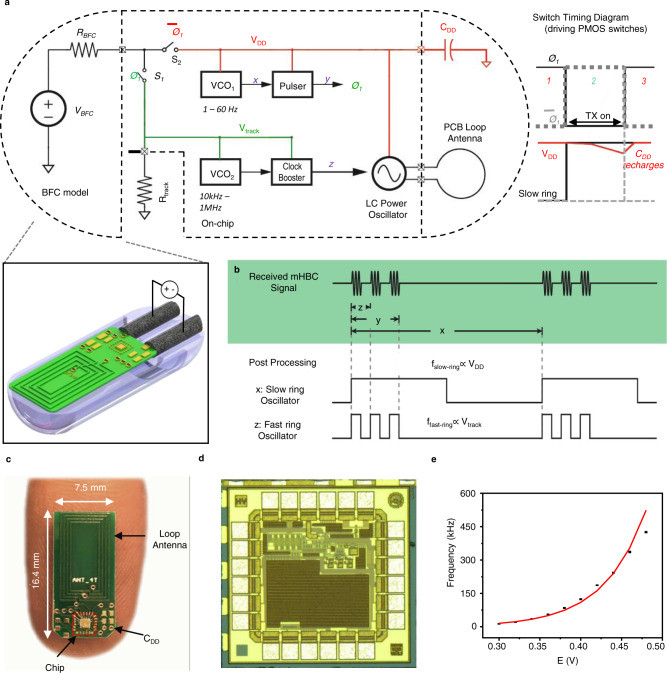
Electrical characterization of the custom integrated circuit. **a** Schematic and operational diagram of the circuit. **b** Timing diagram illustrating pulsed, duty-cycled nature of the circuit, where mHBC pulses of duration *z* occur over a time period *y*, which is a small fraction of the overall inter-transmission interval, *x*. **c** Picture of the prototype electronic board on a human finger. **d** Die microphotograph of the developed chip. **e** Calibration curve of potential to mHBC signal frequency. Data are presented as mean values +/− SD. Error bars represent the standard deviation between multiple circuit boards tested., *n* = 4 independent circuit boards were tested.

The voltage and power derived from the miniaturized glucose BFC are too low to power commercially available wireless transmitters. Larger conventional BFC-powered systems overcome the low-voltage issue by utilizing a DC–DC boost converter to create a higher supply voltage. However, this approach requires bulky power conditioning circuits and introduces additional losses, ultimately exceeding the desired power budget. Herein, we forgo the use of a DC–DC converter and instead developed a custom microchip that can operate directly from the BFC. The integrated circuit was engineered from the ground up to consume less than a microwatt on average (~0.4 µW) via a voltage-to-frequency conversion scheme together with a mHBC transmitter. The schematic, shown in Fig. 2a, operates in two phases, whose timing diagram is shown in Fig. 2b. In phase 1, switch S_2_ is closed, and the BFC is connected to the main system power supply voltage, *V*_DD_, to a trickle-charge energy-buffering capacitor, *C*_DD_. This voltage is used to power a voltage-controlled oscillator (VCO_1_), which varies from 1 to 60 Hz depending on *V*_DD_. On the rising edge of the output of VCO_1_, a pulse is generated (*Ø*_1_), which briefly turns off switch S_2_ while turning on S_1_. This connects the BFC to resistor *R*_track_, generating voltage *V*_track_, which is used to power VCO_2_, generating a 10 kHz–1 MHz signal to modulate an LC-based mHBC power oscillator, powered off of capacitor C_DD_. The developed chip, shown in Fig. 2c, was mounted onto a 16.4 × 7.5 mm circuit board, shown in Fig. 2d, and required connections only to *C*_DD_ and the on-board mHBC antenna. Measurements of the circuit produced the expected quadratic glucose concentration-to-voltage-to-frequency behavior as shown in Fig. 2e.

### Electrochemical design and characterization

**Fig. 3:**
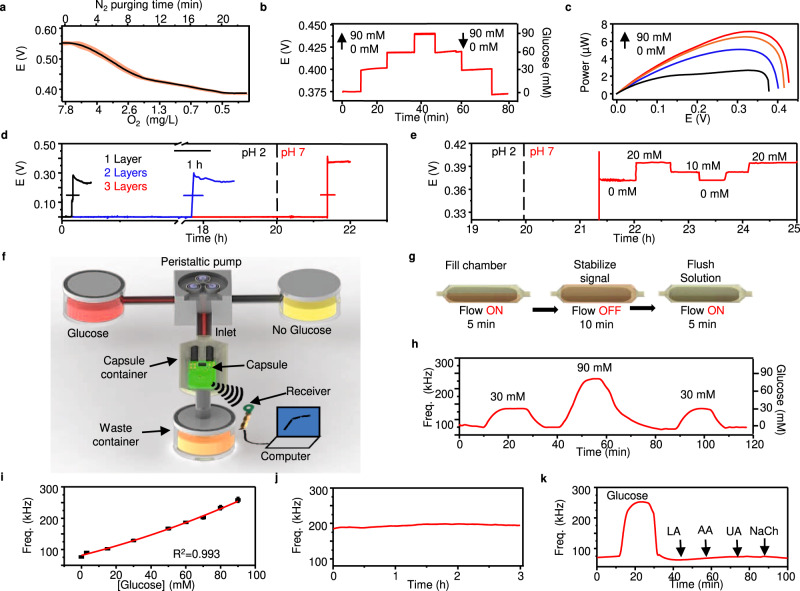
In vitro characterization and simulation of the glucose BFC under close-to-real physiological conditions inside the GI. **a** Measurement of the BFC potential under 200 kΩ load with 30 mM glucose at different O_2_ concentrations during different N_2_ purging times. The red shaded area represents the inter-sample fluctuations, *n* = 4 independent experiments were performed. **b** BFC voltage response under 200 kΩ load toward ascending the descending glucose concentrations (0, 30, 60, and 90 mM). **c** Power of BFC under different glucose concentrations characterized via linear sweep voltammetry. Scan rate, 5 mV s^−1^. **d** pH-sensitive enteric coating optimization with 1,2, and 3 layers of Eudragit® L100 coating in acidic artificial gastric fluid for 20 h followed by neutral artificial intestinal fluid. Data are presented as mean values +/− SD. Horizontal error bars represent inter-sample variations, *n* = 3 independent experiments were performed. **e** Potential of the BFC sensor under load with 3 layers of the enteric coating after activation in response to alternating glucose concentrations (0, 10, and 20 mM). **f** Illustration of the in vitro simulation fluidic system setup. **g** Operating procedure of the fluidic system. **h** Frequency response to alternating glucose concentrations (0, 30, and 90 mM). **i** Calibration curve of glucose concentration to mHBC signal frequency. Data are presented as mean values +/− SD. Error bars represent the standard deviation, *n* = 3 independent experiments were performed. **j** Stability test performed for 3 h in artificial intestinal fluid with 60 mM glucose. **k** Sensor interference test, performed by serial injection of artificial intestinal fluid containing 0 mM glucose, 90 mM glucose, 0.1 mM LA, 0.1 mM AA, 0.1 mM UA, and 3 mM NaCh.

The performance of the BFC has been optimized and calibrated through extensive in vitro characterizations to establish a correlation with the glucose concentration in the small intestine. In the first step, the performance of the BFC was assessed in pH values between 6 and 8, this range of pH levels accounted for the different values of pH in the small intestine^44–46^. The results displayed in Supplementary Fig. 1a showed that the glucose response varies depending on the pH of the solution. In addition, the response of the sensor to the additions of glucose seems to be favorable as the pH decreases. For solutions of pH 6, the sensor provided higher signal increments, while solutions consisting of pH 8 provided lower increments. Such behavior was expected, given that the enzymatic activity is higher between pH values oscillating between 5.5 and 6^47^ Based on the results obtained for the broad range of pH levels tested, lower pH values also resulted in a decrease in the signal coming from the sensor in the absence of glucose, Supplementary Fig. 1b. Next, the system was assessed for its signal generation at a partial pressure of O_2_. We selected pH 6.8 for the evaluation of intestinal O_2_ concentrations due to its similarity to the average pH values reported in the literature for small intestines^48,49^. As shown in Fig. 3a and Supplementary Fig. 2a, with 30 mM glucose, the potential of the BFC decreased from 0.575 to 0.395 upon different nitrogen purging times, such behavior reflected the dependency between oxygen concentration and glucose response from the BFC. The chronoamperometric response of the cathode at variable oxygen levels is depicted in Supplementary Fig. 2b, c. Given that oxygen levels inside the small intestine vary from 2 to 1%^50^, we evaluated the influence of intestinal oxygen on the BFC at different glucose concentrations. The results from Supplementary Fig. 3 showed minimal differences (~4 mV) between the two concentrations of O_2_ tested.

For the temperature studies, we selected a pH level of 6.8 and injected nitrogen gas until the concentration of oxygen was set to 2%. Supplementary Fig. 4 displayed the results obtained by testing the sensor at 35, 37, and 39 °C. For temperature values between 35 and 37 °C, negligible changes in the signals coming from the sensor were observed. Temperature values set at 39 °C showed smaller increments in the signals coming from the sensor compared to the lower temperature values. For further in vitro experiments, we selected 37 °C to account for the physiological temperature effect upon the BFC response. Under such conditions, the potential response of the BFC in 0, 30, 60, and 90 mM glucose solutions demonstrated excellent stability, and reversibility (Fig. 3b). By analyzing a broader range of glucose concentrations (0–110 mM glucose) and applying a quadratic fitting, we obtained a coefficient of determination of 0.991, Supplementary Fig. 5.

The activation of the electronic circuitry for signal transmission requires a power demand of approximately 0.4 µW. Therefore, the corresponding power output at different glucose concentrations (Fig. 3c) also demonstrated that the BFC was able to supply sufficient power in all concentrations, even in the absence of glucose. Such power generated at 0 mM glucose can be attributed to the voltage built between the mediators from the anode and cathode electrodes that integrate the BFC and the oxygen reduction reactions taking place. In the second step, the thickness of the pH-responsive enteric coating was optimized to protect the BFC from the extremely acidic gastric juice (i.e., pH ~ 1.8) upon passage through the stomach until its subsequent dissolution in the neutral intestinal environment (i.e., pH ~ 6.8) which activates the BFC. As shown in Fig. 3d, single and double layers of the pH-sensitive layer of Eudragit® L100 coating were unable to protect the BFC under extended retention (~ 20 h as shown in a prior report^31^) in the acidic artificial gastric fluid, whereas 3 layers assured the full protection of the BFC until its safe transition into the pH-neutral artificial intestinal fluid and subsequent activation. After the dissolution of the enteric coating protection, the BFC demonstrated an excellent dose-dependent response to the changes in glucose concentrations (Fig. 3e).

The passage of the capsule through the GI tract with controlled pH, temperature, and low oxygen profile was simulated using a fluidic system with a peristaltic pump, where a receiver captured the frequency signal in correspondence to the glucose concentration as illustrated in Fig. 3f. Figure 3g depicts the simulation procedure where the flow towards the capsule is turned on and off intermittently to introduce artificial intestinal fluid with different glucose concentrations and purge previous solutions in the system. As a result, the capsule transmitted a reversible response to the alternating glucose concentrations between 0, 30, and 90 mM (Fig. 3h). This setup was used to calibrate the glucose concentration and the transmitted signal frequencies using a quadratic fitting (Fig. 3i), demonstrating good reproducibility between the multiple devices tested. The overall limit of detection and dynamic range of the glucose sensor were 4.656 mM and 3–90 mM, respectively. The stability of the device, evaluated during 3 h of retention in an artificial intestinal fluid containing 60 mM glucose (Fig. 3j), showed minimal changes of 4–7% in the frequency signal of the device (Supplementary Fig. 6). The specific response of the BFC toward glucose was also evaluated through the serial injection of artificial intestinal fluid solutions without glucose, 90 mM glucose, and interfering compounds such as 0.1 mM lactic acid (LA), 0.1 mM ascorbic acid (AA), 0.1 mM uric acid (UA), and 3 mM sodium cholate (NaCh). The results displayed in Fig. 3k demonstrated that glucose-free solutions generated frequency values of approximately 75 kHz, such frequency accounted for the baseline obtained before any compound was injected. After replacing the solution with 90 mM glucose, frequency signals increased up to 250 kHz, followed by the abrupt decay in the signal after flushing the glucose-based solution. The solutions containing the interfering compounds showed negligible cross-reactivity, as the frequency values obtained were close to the ones obtained with the glucose-free solution. To verify the extent of biofouling, we examined the sensor’s response in artificial intestinal fluid containing poly(acrylic acid), mucin, bovine album serum, cholesterol, and phosphatidylcholine, which are the major components of artificial mucus in in vitro settings. By comparing the sensor response after adding glucose to artificial fluid solutions (with and without artificial mucus), small differences (below 10%) were observed, indicating the capsules can operate even in presence of biofouling agents. We did observe a faster change in the signal whenever the artificial solution did not contain the artificial mucus, however, such behavior was expected given the faster diffusion access of glucose to the glucose sensor compartment of the capsule device, Supplementary Fig. 7.

### In situ performance

**Fig. 4:**
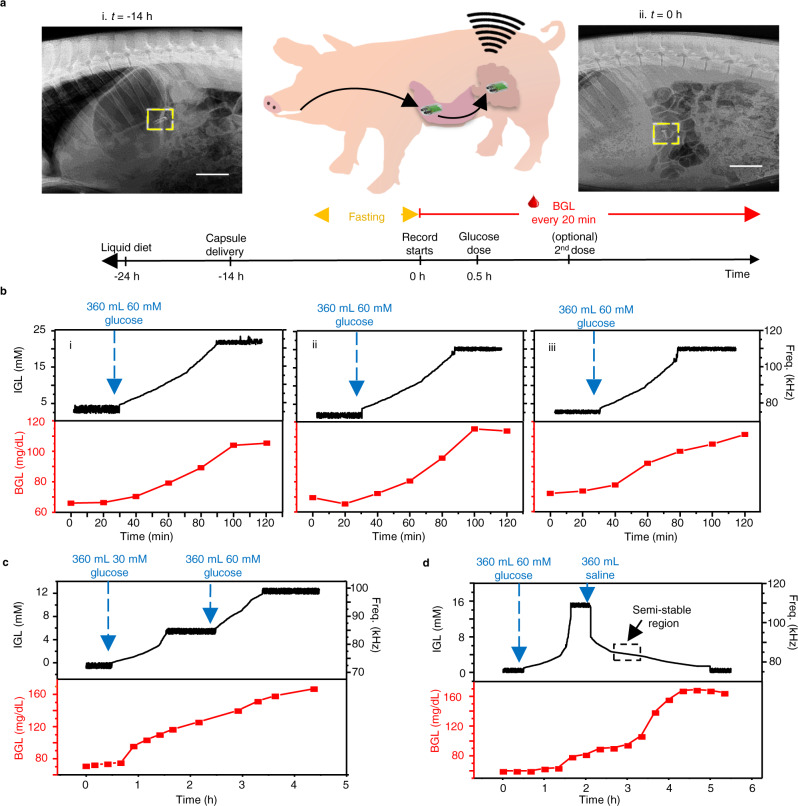
Capsule in situ performance in a porcine model. **a** X-rays images of the pig after oral delivery of the capsule (top) at *t* = −14 h (i) and *t* = 0 h (ii), and the timeline of the experiment protocol (bottom). Scale bar, 5 cm. **b** IGL and BGL monitoring after delivering a 360 mL of 60 mM glucose saline dosage (*t* = 30 min) in 3 independent in situ experiments (i–iii). **c** In situ experiment tracking the IGL and BGL during the sequential delivery of a 360 mL 30 mM glucose saline dosage (*t* = 30 min) followed by a 360 mL 60 mM glucose saline dosage (*t* = 150 min). **d** In situ experiment tracking the IGL and BGL during the sequential delivery of a 360 mL 60 mM glucose saline dosage (*t* = 30 min) followed by a 360 mL glucose-free saline dosage (*t* = 120 min).

The self-powered glucose biosensor was then tested in in situ conditions using a porcine model fed with saline solutions with glucose to simulate food consumption. The capsule location was determined via X-ray imaging, which showed that the capsule was delivered in to the stomach and entered the intestinal domain after 14 h (Fig. 4a and Supplementary Fig. 8). Accordingly, the animal was fed with a liquid diet followed by fasting overnight while delivering the capsule orally *t* = −14 h on the day before the test. After the 14 h, an X-ray image was taken to ensure the capsule moved from the stomach to the small intestine region of the GI tract, *t* = 0 min marks the first capturing of the wireless mHBC signals from the capsule, suggesting the dissolution of enteric coatings. The signals were converted to the real-time intestinal glucose level (IGL) using the calibration obtained from Fig. 3i. Meanwhile, the blood glucose level (BGL) was measured every 20 min using a commercial blood glucose kit. The BGL served as a validation tool to confirm the glucose intake generated a change in the glucose levels of the animal. Figure 4b represents the IGL, and the corresponding BGL in three discrete in situ tests that provide single glucose doses of 360 mL saline with 60 mM glucose delivered orally at *t* = 30 min. Initial low IGL (less than 5 mM) was confirmed by a normal fasting state BGL of ca. 65–70 mg/dL^51^. After administrating the glucose saline solution, the capsule quickly responded with changes in the signal frequency that translates to increases in the IGL until reaching a plateau of 20–22 mM at ca. *t* = 90 min. This observation was supported by the increase in BGL, which also demonstrated the simultaneous glucose absorption from the GI tract to the blood as a part of metabolic functions.

The significantly lower IGL compared to the initial fed dosage can be attributed to the retention of glucose in the stomach or potential dilution due to the presence of intestinal fluid^49,52,53^. The ability of the device to distinguish between different glucose dosages was also evaluated, as shown in Fig. 4c. Here, two dosages of glucose solutions, namely, 360 mL of 30 mM glucose and 360 mL of 60 mM glucose were provided to the animal sequentially at *t* = 30 and 150 min, respectively. The response recorded by the capsule shows that the IGL rose gradually and plateaued at 6 mM in response to the initial glucose dosage, followed by the secondary dosage that further boosted the IGL to 12 mM. The BGL, on the other hand, demonstrated a slightly delayed, monotonous increase from 70 mg/dL at *t* = 0 to ca. 170 mg/dL over 270 min. The influence of glucose-free intake was also studied via the sequential delivery of 360 mL of 60 mM glucose at *t* = 30 min followed by 360 mL of glucose-free saline solution at *t* = 120 min. As demonstrated in Fig. 4d, the first dose of the solution increased the IGL approximately to 16 mM. The introduction of the glucose-free solution led to a slightly delayed yet sharp signal decrease suggesting the dilution of the IGL, which eventually dropped to near 0 mM after a period of 3 h at *t* = 300 min. It is worth noticing that around *t* = 180 min, the capsule reported a semi-stable IGL of 4–6 mM as the response to the equal dilution of glucose intake (to 30 mM), similar to the result shown in Fig. 4c. This was followed by a gradual decrease in the IGL and a continuous increase in BGL, suggesting the continuous absorption of glucose from the GI tract to the bloodstream^54^. The device was capable of operating without the ingestion of any solution. As shown in Fig. 4b–d, the initial 30 min of each set of experiments recorded the signals without the administration of any glucose or glucose-free solution. Since the intestinal fluid is always present inside the small intestine (even during fasted state) in volumes above 100 mL^53^, such amounts of fluid are sufficient to interact with the glucose sensor to make the device operate. The submergence of the capsule inside the gut was also facilitated by the periodic contraction and mixing motions, which have kept the capsule always in the transverse orientation exposing the electrodes to the fluid either on the posterior or anterior side^55,56^. Improper interaction between the intestinal fluid and the capsule device would result in lower values of voltage outside the range required for data transmission, Supplementary Fig. 9.

Given that our in vitro experiments did not include any mechanical motion, we hypothesize that the higher noise observed during the plateaus (Supplementary Figs. 10–14) could be caused by the migrating motor complex (MMC) and the lower-power noise could arise from the intestinal movements present during digestion. The MMC is characterized by irregular and regular contractions that are present during the fasted state, originating in the stomach and small intestine^57^. After feeding, the MMC is suppressed, and digestion takes place. During digestion, mechanical motion occurs through peristaltic and segmentation movements inside the small intestine, which mixes and propels solid-state and liquid bolus^56,58^. The administration of the sugar-based and sugar-free solutions, which corresponds to the intervals of raising and lowering steps in Fig. 4b–d, might lead to the suppression of the MMC. This could cause the potentially movement-induced noise to be suppressed, resulting in significantly less noise during the raising and lowering steps. Previous studies have found similar behavior, in which the electrical activity in the small-intestine of rats indicated that the MMC was suppressed after the rats were fed a glucose-based solution^59^. This behavior is dependent on the number of calories and the nature of food provided to the subject. For instance, fats can cause longer disruption of the MMC than carbohydrates and proteins^60^. For our experiments, the plateau domains observed after feeding the solutions to the pig could be attributed to the recovery of the MMC after a certain period, though this will be investigated in more detail in future work. Additionally, based on the relative rate of change of glucose observed in the GI tract, it’s also likely that our sampling rate is simply too high—we do not need to sample so frequently to get a good picture of glucose dynamics in the system. Data presented in Supplementary Fig. 15 show the response curves under a low pass filter of Fig. 4b–iii. Here, the higher-frequency noise is eliminated, without compromising the underlying concentration data. It can also be shown that although closely related, the ingested, intestinal, and blood glucose levels can differ significantly as a result of digestion and glucose homeostasis processes affected by individual physiological parameters^61^. In addition, our in situ experiments were performed under scenarios without solid-food content, the data presented in Supplementary Fig. 16 shows that solid food can generate a decrease in the response resulting from the sensor. Therefore, such scenarios are worthy of separate investigations in future work.

## Discussion

In this work, we demonstrated a self-powered, battery-free ingestible biosensing capsule for in situ, real-time intestinal glucose monitoring in living subjects. To activate the electronic circuitry inside the capsule, the integrated BFC, which also acts as the sensor, scavenges sufficient power for an energy-mHBC wireless circuit. This work addressed multiple existing challenges in capsule-type ingestible devices, including the elimination of batteries, miniaturization of electronics, signal transmission through thick biological tissues, and protection of biosensors against harsh in situ environments. During in vitro simulations, the BFC sensor showed high specificity toward glucose, while solutions containing the interfering compounds showed negligible cross-reactivity. By analyzing the rate of change during the extended monitoring of the frequency data, minimal changes were observed in the signals recorded. The in situ experiments performed on pigs showed that the difference in glucose concentration from the orally delivered solutions generated differences in the continuous data collected, implying the successful detection of glucose from the small intestine.

Since there are no other ways to comfortably measure real-time intestinal glucose concentrations, the development of an ingestible device capable of monitoring the glucose levels inside the small intestine can furtherly expand the scope of in situ real-time monitoring of glucose and potentially provide a more comprehensive understanding of metabolic interactions and kinetic models for glucose absorption in people with normal conditions, pregnancy, or diabetes^62^. Also, such technology could play a key role in the diagnosis of malabsorptive conditions, such as chronic pancreatitis and dumping syndrome, e.g., by analyzing if complex carbohydrate meals are sufficiently broken down to glucose and the gastric emptying time required from food bolus to relocate in the small intestine. Although current studies involving glucose diagnosis, chronic pancreatitis, and dumping syndrome make use of glucose tolerance tests^63^, radioactive tracers^64^, and urine sample collection^65^, such approaches cannot provide real-time analysis of the absorption rates, lack of activity time, or require a large amount of radioactive material during metabolic rate diagnosis^66,67^.

Future work will be centered on addressing limitations and expanding the functionality of the capsule sensing platform. For instance, the use of the biosensor on porcine subjects without anesthesia; with procedures involving solid food ingestion where solid particles may partially block the sensor surface and require a modified porous membrane to prevent sensor fluctuations, integration of functionalities such as tracking of oxygen concentration, temperature, and pH to further calibrate the capsule toward reliable glucose monitoring. Our current prototype was limited to glucose monitoring in solution, however, the BFC generated sufficient power to activate the data transmission module. Therefore, such power could also be used to power other sensor front-ends for potentiometric sensing, or even possibly for amperometric sensing. Thus, with advances in integrated circuits research, it may be possible to add non-self-powered sensors (such as pH, oxygen, electrolytes, and foreign drugs) to the platform by exploiting the excess energy available from the glucose BFC.

In addition, to allow easier passage of the capsule through the GI tract, the dimensions of the device will be reduced to those used for commercial ingestible devices^68^ by fabricating smaller sensors and electronic components. The next-generation energy-autonomous GI tract biosensing system tracking various biochemicals such as electrolytes, lactate, vitamins, fatty acids, and foreign drugs could be of tremendous interest in obtaining a more comprehensive picture of the gut environment and could promote the diagnosis of various related symptoms and diseases.

## Methods

### Ethical statement

The animal experiments were conducted following the protocols approved (S19197) by the Institutional Animal Care and Use Committee (IACUC) Office at the University of California, San Diego.

### Materials and reagents

Polyvinylidene difluoride (PVDF), N-Methyl-2-pyrrolidone (NMP), Mesoporous carbon (MC), 7,7,8,8-tetracyanoquinodimethane tetrathiafulvalene (TTF-TCNQ), isopropanol alcohol (IPA), glucose oxidase (GOx) from aspergillus niger, chitosan, toluene, acetic acid, polyvinyl alcohol (PVA), ethanol (EtOH), potassium chloride (KCl), potassium dihydrogen phosphate (KH_2_PO_4_), Potassium phosphate dibasic (K_2_HPO_4_), sodium bicarbonate (NaHCO_3_), sodium chloride, (NaCl), magnesium chloride (MgCl_2_), ammonium chloride (NH_4_Cl), uric acid, hydrochloric acid, d -(+)-glucose, sodium cholate, calcium chloride, mucin, sodium hydroxide (NaOH), lactic acid, ascorbic acid, poly(acrylic acid) (PAA), mucin, bovine serum albumin (BSA), cholesterol and phosphatidylcholine (PC) were purchased from Sigma-Aldrich (St. Louis, MO, USA). Carboxyl-functionalized multi-walled carbon nanotubes (MWCNT‐COOH, *Ø* = 10–20 nm, 10–30 µm length, >95% purity) were purchased from Cheap Tubes Inc. Nickel foam (1 mm thickness) was purchased from Amazon (brand: Futt). Polyurethane (PU) (Tecoflex SG-80A) was obtained from Lubrizol. Styrene-ethylene/butylene-styrene-maleic anhydride-graft (SEBS-MA) was obtained from Kraton^TM^. Bilirubin Oxidase (BOD, > 1.2 U mg ^−1^) was obtained from Amano Enzyme. Stainless steel wire (421 µm) was purchased from Master Wire Supply. 2,2′-azino-bis (3-ethylbenzothiazoline-6-sulfonic acid) (ABTS) was purchased from Alfa aesar (India). Clear silicone was purchased from Amazon (Brand: J-B Weld). Eudragit® L100 was obtained from Evonik. Barium Sulfate (BaSO_4_) was purchased from Amazon (Brand: HiMedia). Ensure® and Simple Truth Organic^TM^ Unsweetened Applesauce were purchased from Amazon.

### Chip manufacturing and assembly

The microchip was fabricated in a 180 nm CMOS process and mounted onto a printed circuit board (PCB) produced using FR-4 material. The copper traces on the PCB were plated with gold to facilitate wire bonding to the bond pads on the microchip. The mHBC antenna was designed using copper traces on the PCB itself, and *C*_DD_ ceramic capacitor was soldered to the surface of the PCB.

### Electrical characterization

The integrated circuit was first tested in a benchtop environment, where power supplies (Keithley 2602, Tektronix, Beaverton, OR), were used to simulate the BFC, and an oscilloscope (Keysight InfiniiVision MSOX 4024A, Santa Rosa, CA) was used to capture the voltage-to-frequency calibration curve. A wireless mHBC receiver was built using minicircuits (Brooklyn, NY) components to down-convert the wirelessly received signal to baseband prior to frequency extraction on an oscilloscope.

### Capsule case fabrication

First, the design of the capsule case was developed using SolidWorks software. Two pieces comprise the capsule case (top and bottom); the diameter of the capsule device was 0.9 cm, of which each electrode inside the capsule occupied 0.35 cm. The length of the capsule was 2.6 cm. The design of the capsule was made using SolidWorks® (x64 2016). The capsule was 3D-printed using an Anycubic Photon S–UV Resin DLP 3D Printer. After the printing, both capsule pieces were immersed in an IPA-containing baker to remove the resin excess by sonication in an ultrasonic bath for 15 min. Afterward, both capsule parts were cured using a UV-Lamp (350 nm) for 10 min.

### Electrode fabrication and enzymatic modification

A porous Nickel foam piece (Ni), cut into 1 × 0.5 cm pieces, served as a base material for both anode and cathode. Each piece was folded as a spiral, creating a cylinder-shaped tube of 0.35 cm diameter and 0.6 cm length, 0.2 cm section of which was dedicated to introducing a stainless-steel (ss) wire. The Ni-ss connection was insulated with SEBS-MA (2 g/10 mL in toluene), dried at 60 °C, and the electrodes were trimmed to the final length of a 0.4 cm length. Both electrodes were dip-coated 10 s in a customized carbon-based slurry, consisting of 0.15 g MWCNT-COOH, 0.15 g MC and 0.8 g SEBS-MA in 14 mL of toluene, which was thoroughly sonicated for 10 min, using a 300 W ultrasonic probe processor (Ultrasonics), set on 30% power, (2 s pulses, 50% duty cycle). After finishing the dip-coating step, the electrodes were dried at 60 °C for 5 min. This step was repeated until the Ni material was completely covered with the carbon slurry.

Enzymatic modification: the anode was modified by immersing the electrode into TTF-TCNQ (5 mg/mL in NMP), PVDF (10 wt% in NMP)- MC (10 mg/mL) containing solution (previously sonicated under the same conditions as the MWCNT-SEBS-MA solution), for 15 s and left to dry at 60 °C. The cathode was modified following the same steps but using ABTS (5 mg/mL in NMP), PVDF (10 wt% in NMP)- MC (10 mg/mL) containing solution. After the drying process, the color of the anode and cathode changed from black to dark yellow and blue, respectively. The excess of mediator was removed by running repetitive cyclic voltammetry scans from −0.2 to 0.5 V (for the anode) and from 0 to 0.7 V (for the cathode) in 0.1 M potassium phosphate buffer (PBS) pH 7.3. The repetitive scans were repeated until the cyclic voltammogram for each electrode remained constant. Scan rate: 30 mV/s.

Both anode and cathode were modified with GOx (30 mg/mL) and BOD (30 mg/mL) enzyme solutions in PBS pH 7.3, respectively, wetting the hydrophobic surface of the electrodes with 15 µL of IPA before biocatalysts application. Both electrodes were allowed to dry for 1 h at room temperature, and the process was repeated once again. Next, 2 layers of 15 μL of chitosan (0.5 wt% in 0.1 M acetic acid) were drop-casted on each electrode to stabilize the biocatalyst layers on the surface. The modified electrodes were stored at 4 °C overnight.

### In vitro characterization

All in vitro studies without the flow system were carried out using a Metrohm Autolab potentiostat/galvanostat and Nova 2.0 software. The ss ends of each electrode were connected to the equipment and to a 200 kΩ resistor during the complete test. Experiments from Fig. 3a and Supplementary Figs. 1–3 were performed at room temperature. Experiments from Fig. 3b–e and Supplementary Fig. 5 were performed at 37 °C. Nitrogen gas was injected until the concentration of oxygen was set to 2%. Oxygen concentration was measured using a dissolved Oxygen Meter (RCYAGO) purchased from Amazon.

Enteric coating modification for in vitro optimization test: Each electrode was placed in a 4 mm-diameter and 8mm-length plastic tube and sealed with two subsequent dosages of 7 and 3 µL of PVA solution (5 wt% in deionized water) on the active area side of the electrodes, drying the layers for 2 h at room temperature after each application. Next, 5 µL of the pH-responsive Eudragit ® L100 polymer (4 wt% in EtOH) were drop-casted on top of the PVA layer and left to dry at room temperature, creating a pH-responsive enteric coating layer. This step was repeated twice to create two layers or three times to create three layers.

Artificial gastric fluid preparation: the artificial gastric juice was prepared by mixing specific volumes of KCl (46.72 g/L), KH_2_PO_4_ (68 g/L), NaHCO_3_ (84 g/L), NaCl (120 g/L), MgCl_2_ (30 g/L), and NH_4_CL (27.28 g/L) in deionized water^69^ and the acidity was corrected to pH = 1.8 using a solution of 3 M HCl in deionized water.

Artificial intestinal fluid preparation: the artificial intestinal juice was prepared by a specific volume of solutions of KCl (46.72 g/L), KH_2_PO_4_ (68 g/L), NaHCO_3_ (84 g/L), NaCl (120 g/L), MgCl_2_ (30 g/L), and in deionized water, as was previously reported^69^. The pH of the solution was adjusted to 6.8 using 1 M HCl or 1 M NaOH in deionized water. For biofouling experiments, we adapted a recipe used in a previous work^70^. The intestinal fluid solution was mixed with 0.3% (w/v) of PAA, 3% (w/v) mucin, 0.6% (w/v) of BSA, 0.36% (w/v) of cholesterol, and 0.18% (w/v) of PC.

### Encapsulation process for flow system and in situ experiments

The ss current collector of each BFC electrode was soldered to the electronic board using liquid soldering flux. A heat gun set at 90 °C was used to melt a 1 × 1.5 cm PU sheet to insulate the electronic board after soldering the electrodes to the electronic board, followed by encapsulation into the 3D-printed capsule and filling the void volume with 50 mg of BaSO_4_. The junction of the hollow capsule shells was closed and insulated using a thin layer of commercial biocompatible waterproof silicone elastomer and dried for 2 hours. An additional insulating layer of PU was used to protect the capsule, applying 90 °C gun heating on a 1.5 × 0.6 cm sheet. The insulated BFC capsule was stored at 4 °C until its usage.

### In vitro characterization using the flow system

With the assistance of a peristaltic pump, solutions of a determined glucose concentration were injected using a flow rate of 3 mL/min for 5 min. During this time, the solution covered the inlet and capsule container. Afterward, the peristaltic pump was stopped for 10 min to allow complete contact between the BFC electrodes and the solution, and data was recorded wirelessly. After recording, the peristaltic pump was activated again for 5 min, washing the corresponding solution in an external waste container. All the experiments were performed at 37 °C. Nitrogen gas was injected until the concentration of oxygen was set to 2%.

### Wireless signal acquisition process from the capsule-based device

The in vitro studies with the flow system and in situ experiments with animals were carried out using an Oscilloscope Keysight Infinii version MSOX4024A. Data obtained from the device was recorded every 5 s. By implementing a VCO, the integrated circuit can effectively convert the voltage from the BFC corresponding to respective glucose concentration to frequency data. This frequency data is wirelessly transmitted using on–off keying (OOK) modulation, an on-chip power oscillator, and an off-chip 4-turn PCB antenna through mHBC. The mHBC focuses on the magnetic resonant coupling that leverages the body’s low magnetic field losses and effectively turns the body into a dielectric waveguide or magnetic bubble. This is ideal for low-power on-body sensor networks interfacing with other on-body smart electronics. The data is then received by a receiver system connected to a laptop computer equipped with a custom application for real-time data processing and visualization. The frequency data is converted back to glucose concentration with the use of calibration curves.

### Animal in situ experimental procedures

The experiments were conducted following the protocols approved by the Institutional Animal Care and Use Committee (IACUC) Office at the University of California, San Diego. In situ tests were performed in farm pigs (no preference in sex) between three to eight months and weighing between 42 and 50 kg.

#### Anesthesia

Anesthesia machine with a precision isoflurane vaporizer/O_2_ isoflurane (1–3% in O_2_) flow meter was used in all in vivo experiments, in addition to 0.05 mg/kg atropine, 2 mg/kg xylazine, and 20–30 mg/kg ketamine, injected intravenously, to anesthetize the animal before the experiment commence. A pulse oximeter was used to monitor the subject’s heart rate (HR) and oxygen saturation (SpO2).

#### Intubation

After the animal was anesthetized, the animal was subjected to oral gavaging using an esophageal tube (10 mm, diameter). The tube splinted the pharynx and oral esophagus was used to administrate the capsule device, prevent its physical damage (i.e., by chewing), and deliver liquid feeding dosages during the experiment. Supplemental heating was provided to keep the pig warm.

#### X-ray imaging

images were taken using a standard X-ray tabletop machine to provide a lateral image after delivery of the capsule inside the stomach and before the start of the signal recording.

#### Saline solutions for in situ experiments

the solution was prepared by mixing 32.3 mM of K_2_HPO_4_, 32.7 mM of KH_2_PO_4,_ and 45 mM of NaCl in deionized water. A few milliliters were left to adjust the pH to 6.8 using 2 M HCl and 2 M NaOH. After that, the volume was adjusted to the final volume.

#### Protocol for in situ experiments with pigs

before the measurements (−24 h), the animal was kept in a liquid diet using Ensure® and Simple Truth Organic^TM^ Unsweetened Applesauce for 24 h. Ten hours later (−14 h), the animal used for experiments was anesthetized to place the capsule device inside the stomach using the esophageal tube. Next, an X-ray image was taken to confirm the delivery of the capsule inside the stomach. After that, the anesthesia was removed from the animal. Subsequently, the animal was left doing overnight fasting of 8 h. Fourteen hours later (0 h), the anesthesia was applied again to the animal to obtain a second X-ray image, confirming the relocation of the capsule in the intestines. A couple of minutes later, signal recording took place without any glucose intake for 30 min. Simultaneously blood samples were obtained every 20 min using an Accu-Chek® Aviva glucose meter during the whole experiment. After capturing the signal without glucose intake, glucose feeding steps of 360 mL of saline solutions with different concentrations were provided to the animal.

### Statistics and reproducibility

In some of the in vitro experiments, standard deviation (SD) was used to evaluate the reproducibility of the devices. A sample size of *n* = 3 was used. No statistical method was used to predetermine sample size. No data were excluded from the analyses. During in situ experiments, we do not perform any statistical analysis. The investigators were not blinded to allocation during experiments and outcome assessment.

### Reporting summary

Further information on research design is available in the Nature Portfolio Reporting Summary linked to this article.

## Supplementary information

Supplementary information

Reporting Summary

## Data Availability

All data generated that supports the findings of this study are available in the source data file provided with the manuscript. Source data are provided with this paper.

## Source data

Source data file
