# Supplementary material for: A self-powered ingestible wireless biosensing system for real-time in situ monitoring of gastrointestinal tract metabolites
Source: Nat Commun. 2022 Dec 1;13:7405. doi: 10.1038/s41467-022-35074-y (PMC9715945; doi:10.1038/s41467-022-35074-y)
Supplement: Supplementary file 1 — Supplementary information [file 41467_2022_35074_MOESM1_ESM.pdf]

A self-powered ingestible wireless biosensing system for real-time in-situ monitoring of gastrointestinal tract metabolites

Ernesto De la Paz[1†], Nikhil Harsha Maganti[2†], Alexander Trifonov[1†], Itthipon Jeerapan[1†], Kuldeep Mahato[1], Lu Yin[1], Thitaporn Sonsa-ard[1], Nicolas Ma[1], Won Jung[1], Ryan Burns[2], Amir Zarrinpar[3,4,5], Joseph Wang[1]* and Patrick P. Mercier[2]*

[1]Department of Nanoengineering, University of California San Diego, La Jolla, California 92093, USA.

[2]Department of Electrical and Computer Engineering, University of California San Diego, La Jolla, California 92161, USA.

[3]Division of Gastroenterology, University of California San Diego, La Jolla, California 92093, USA.

[4]VA San Diego Healthcare System, La Jolla, California 92161, USA

[5]Center for Microbiome Innovation, University of California San Diego, La Jolla, California 92093, USA.

[†]These authors contributed equally to this work

*e-mail: pmercier@ucsd.edu, josephwang@ucsd.edu

Table of Contents

Supplementary Fig.1. Performance of the biofuel cell sensor under different pH values inside the small intestine ... 3

Supplementary Fig. 2. Oxygen dependency study ... 3

Supplementary Fig. 3. Performance of the biofuel cell sensor under different oxygen values inside the small intestine ... 4

Supplementary Fig. 4. Performance of the biofuel cell sensor under different temperature values inside the small intestine ... 4

Supplementary Fig. 5. BFC glucose calibration curve at 15 min N_2 purging ... 5

Supplementary Fig. 6. Percentage of change during stability experiment ... 5

Supplementary Fig. 7. Biofouling study with artificial mucus ... 6

Supplementary Fig. 8. Retention time of the capsule device inside the stomach ... 6

Supplementary Fig. 9. Different angles of contact between capsule device and artificial intestinal fluid ... 7

Supplementary Fig. 10. High pass filtered data from Figure 4b-i from the manuscript ... 7

Supplementary Fig. 11. High pass filtered data from Figure 4b-ii from the manuscript ... 8

Supplementary Fig. 12. High pass filtered data from Figure 4b-iii from the manuscript ... 8

Supplementary Fig. 13. High pass filtered data from Figure 4c from the manuscript ... 9

Supplementary Fig. 14. High pass filtered data from Figure 4d from the manuscript ... 10

Supplementary Fig. 15. Low pass filtered data from Figure 4b-iii from the manuscript ... 10

Supplementary Fig. 16. Solid food effect on the BFC sensor ... 11

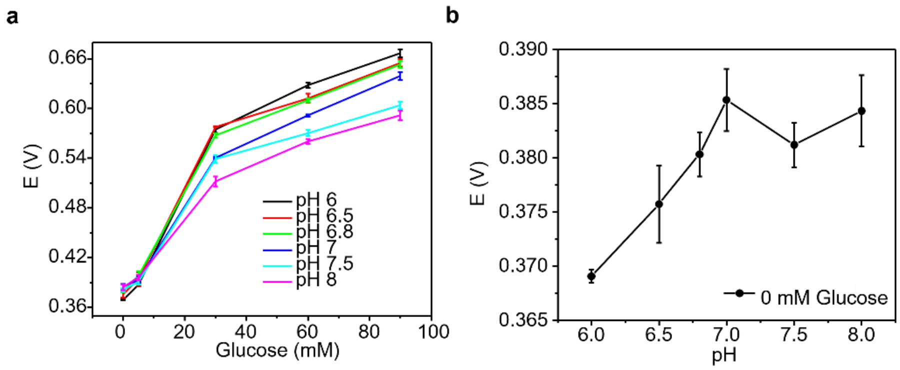

Supplementary Fig. 1. Performance of the biofuel cell sensor under different pH values inside the small intestine. a, The response of the biofuel cell sensor was tested at 0, 3, 30, 60, and 90 mM glucose under the physiological range of pH levels inside the small intestine. b, Voltage signals at different pH levels in the absence of glucose. Data are presented as mean values +/- SD. Error bars represent the standard deviation between multiple sensors tested, *n* = 3 independent experiments were performed.

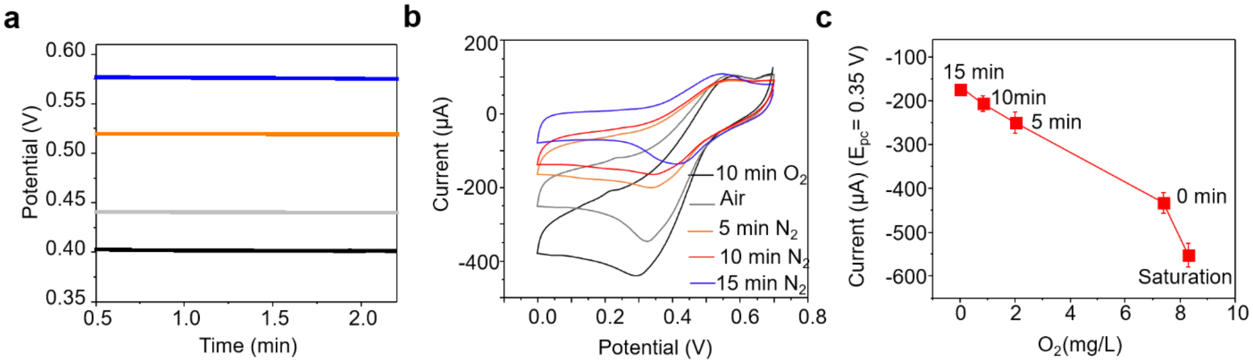

Supplementary Fig. 2. Oxygen dependency study. a, Voltage at constant load of 200 kΩ at variable N_2 purging times: 30 mM glucose at normal air conditions (blue solid line), after 5 min of N_2 purge (orange line), 10 min of N_2 purge (gray line), and 15 min of N_2 purge (black line). b, Cathodic cyclic voltammograms at different O_2 concentrations in the electrolyte purging N_2 for 15 min (blue), 8 min (red), 5 min (orange), 0 min (gray), or O_2 saturation bubbling for 10 min (black). c, Calibration curve derived from cathodic current peaks (E_{pc}) of b from the cyclic voltammograms at 0.35 V vs. Ag/AgCl. Data are presented as mean values +/- SD. Error bars represent the standard deviation between multiple sensors tested, *n* = 3 independent experiments were performed.

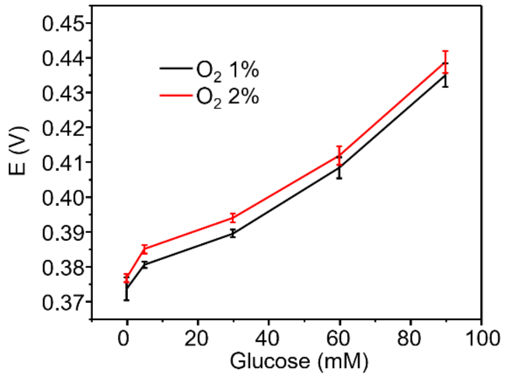

Supplementary Fig. 3. Performance of the biofuel cell sensor under different oxygen values inside the small intestine. The response of the biofuel cell sensor was tested at 0, 3, 30, 60, and 90 mM glucose under the physiological range of oxygen (O_2) levels inside the small intestine. The pH value of the solutions was set to 6.8. Data are presented as mean values +/- SD. Error bars represent the standard deviation between multiple sensors tested, *n* = 3 independent experiments were performed.

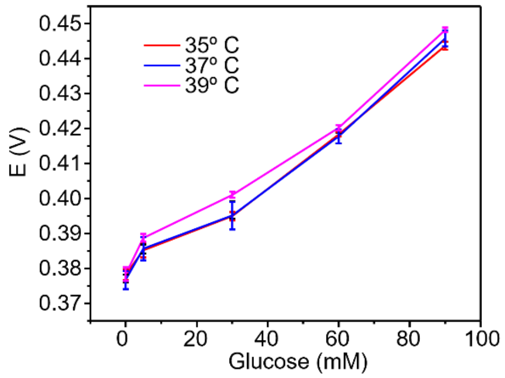

Supplementary Fig. 4. Performance of the biofuel cell sensor under different temperature values inside the small intestine. The response of the biofuel cell sensor was tested at 0, 3, 30, 60, and 90 mM glucose under the physiological range of temperature levels inside the small intestine. The pH value of the solutions was set to 6.8. The oxygen concentration was set to 2%. Data are presented as mean values +/- SD. Error bars represent the standard deviation between multiple sensors tested, *n* = 3 independent experiments were performed.

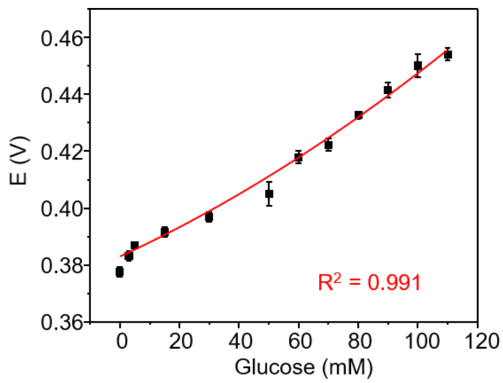

Supplementary Fig. 5. BFC glucose calibration curve at 15 min N_2 purging. The voltage response of the BFC towards glucose was tested by increasing the glucose concentration from 0 to 110 mM. A quadratic regression (red curve) was applied to the experiment to obtain the quadratic equation and coefficient of determination (R^2). Data are presented as mean values +/- SD. Error bars represent the standard deviation between multiple sensors tested, *n* = 3 independent experiments were performed.

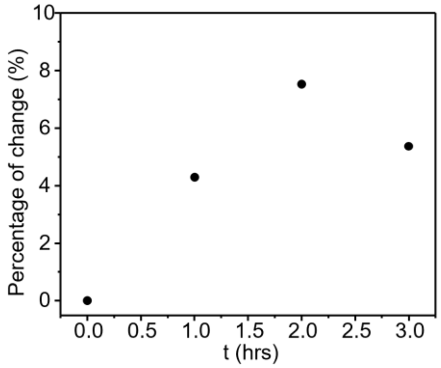

Supplementary Fig. 6. Percentage of change during stability experiment. The frequency change obtained from the BFC while its immersion in 60 mM glucose was evaluated for 3 hours. To calculate the percentage of change, frequency values were taken at 1, 2, and 3 hrs. of starting the experiment. The difference between such values and the initial value at 0 hrs. was divided by the frequency point at 0 hrs. and multiplied by 100.

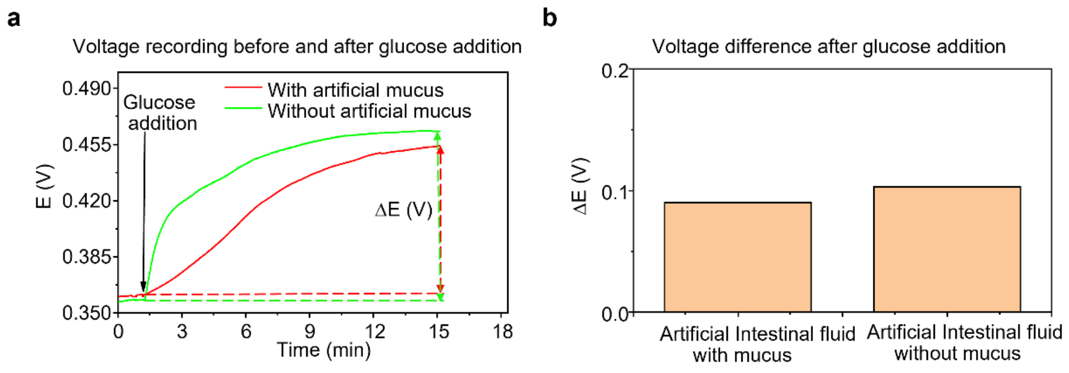

Supplementary Fig. 7. Biofouling study with artificial mucus a, Signal recording of the biofuel cell sensor in the presence of artificial mucus (red curve) and in the absence of artificial mucus (green curve). After 90 sec of recording, glucose was injected into each solution. The voltage difference (ΔE) was calculated by the difference between the signal after glucose injection and before glucose injection. b, Comparison of the voltage difference obtained in the presence and absence of artificial mucus.

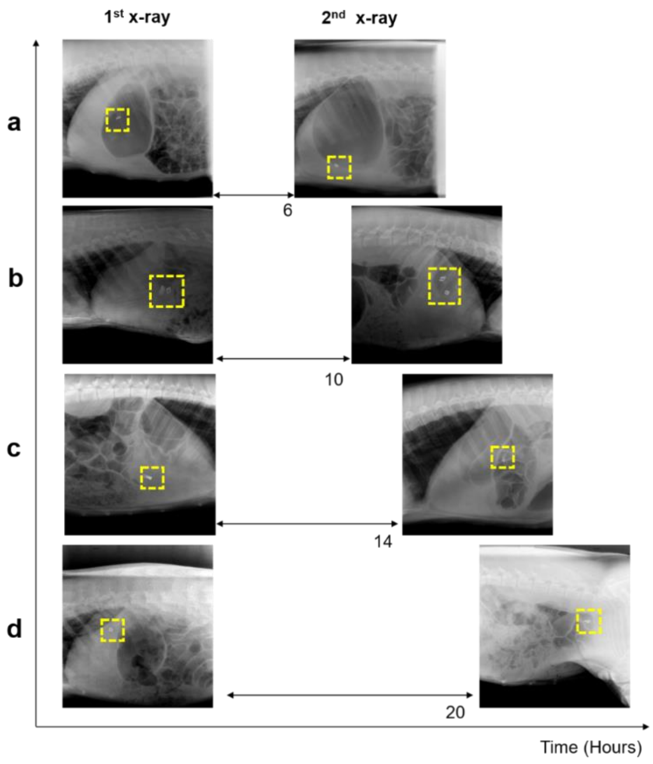

Supplementary Fig. 8. Retention time of the capsule device inside the stomach. a, x-ray comparison before and after 6 hours of delivery of capsule inside the stomach. b, x-ray comparison before and after 10 hours of delivery of capsule inside the stomach. c, x-ray comparison before and after 14 hours of delivery of capsule inside the stomach. d, x-ray comparison before and after 20 hours of delivery of capsule inside the stomach.

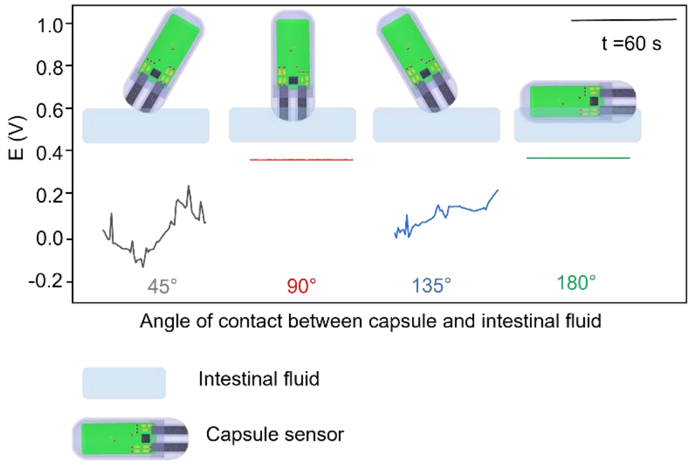

Supplementary Fig. 9. Different angles of contact between capsule device and artificial intestinal fluid. The capsule device with the BFC sensors integrated was immersed in artificial intestinal fluid at 45, 90, 135, and 180°. The voltage signal obtained was recorded at each angle of contact.

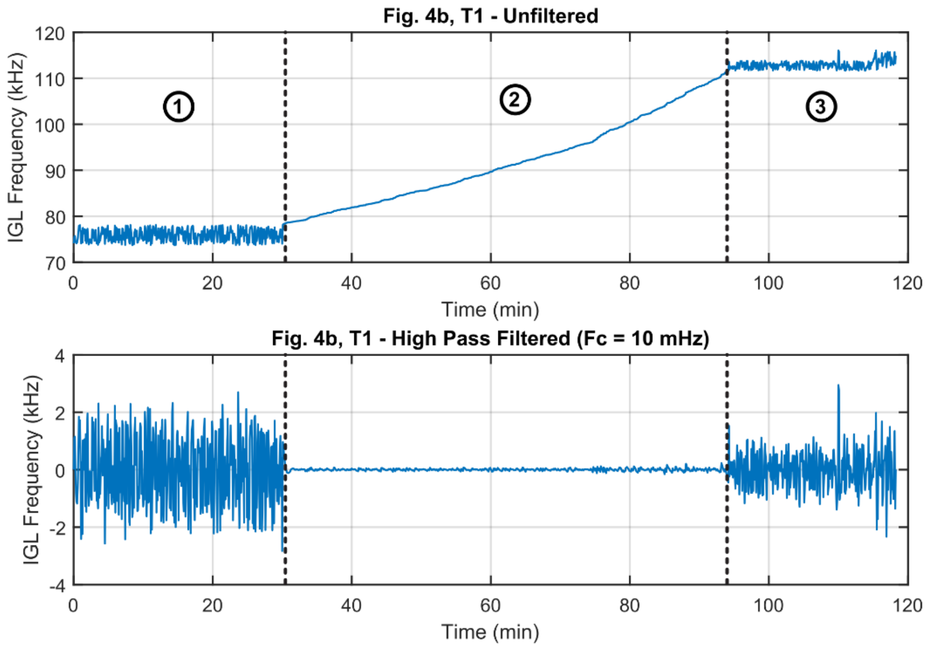

Supplementary Fig. 10. High pass filtered data from Figure 4b-i from the manuscript. (top) Data unfiltered. Domains 1 and 3 represent the plateau sections. Domain 2 represents the raising step. (bottom) High pass filtered (Fc = 10 mHz).

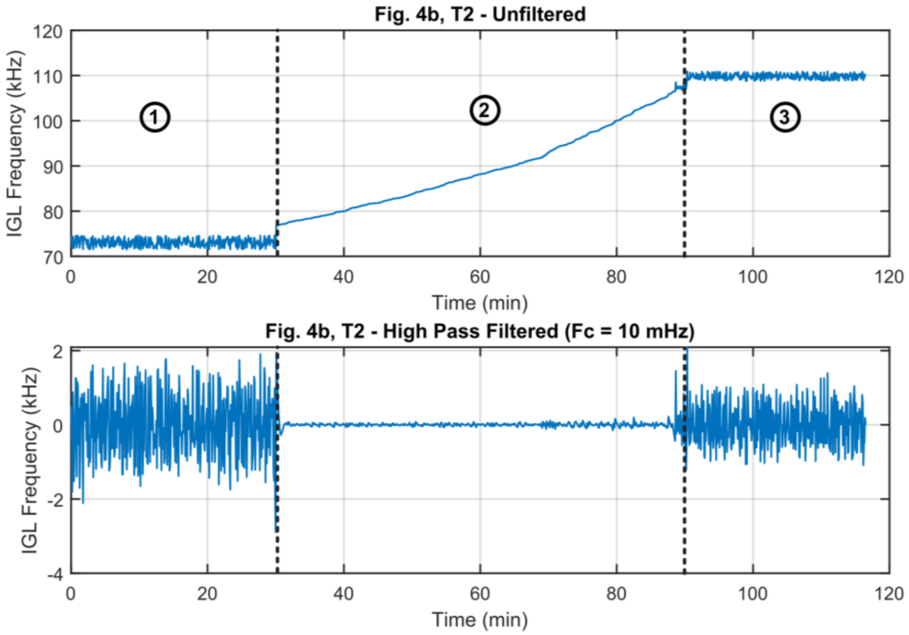

Supplementary Fig. 11. High pass filtered data from Figure 4b-ii from the manuscript. (top) Data unfiltered. Domains 1 and 3 represent the plateau sections. Domain 2 represents the raising step. (bottom) High pass filtered (Fc = 10 mHz)

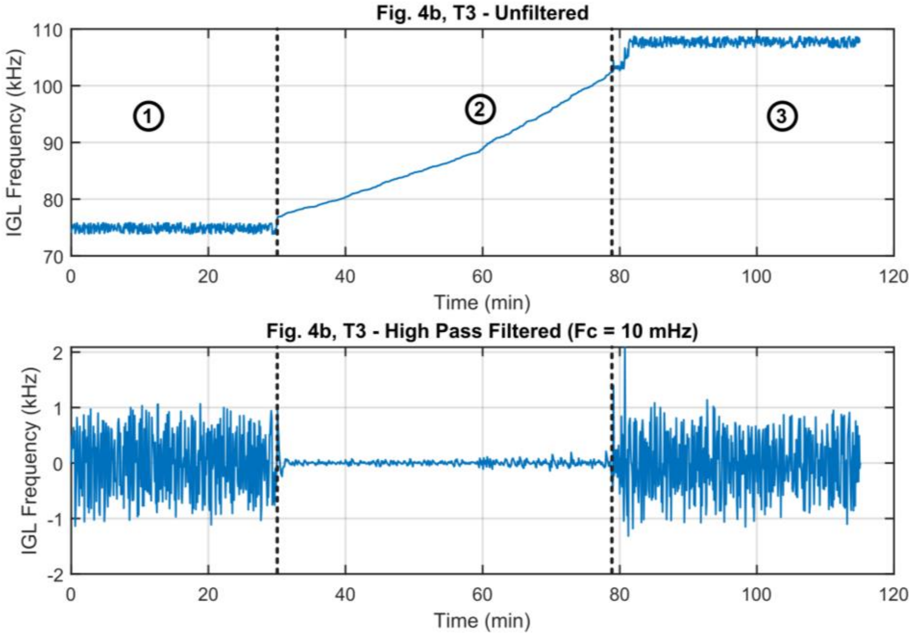

Supplementary Fig. 12. High pass filtered data from Figure 4b-iii from the manuscript. (top) Data unfiltered. Domains 1 and 3 represent the plateau sections. Domain 2 represents the raising step. (bottom) High pass filtered (Fc = 10 mHz)

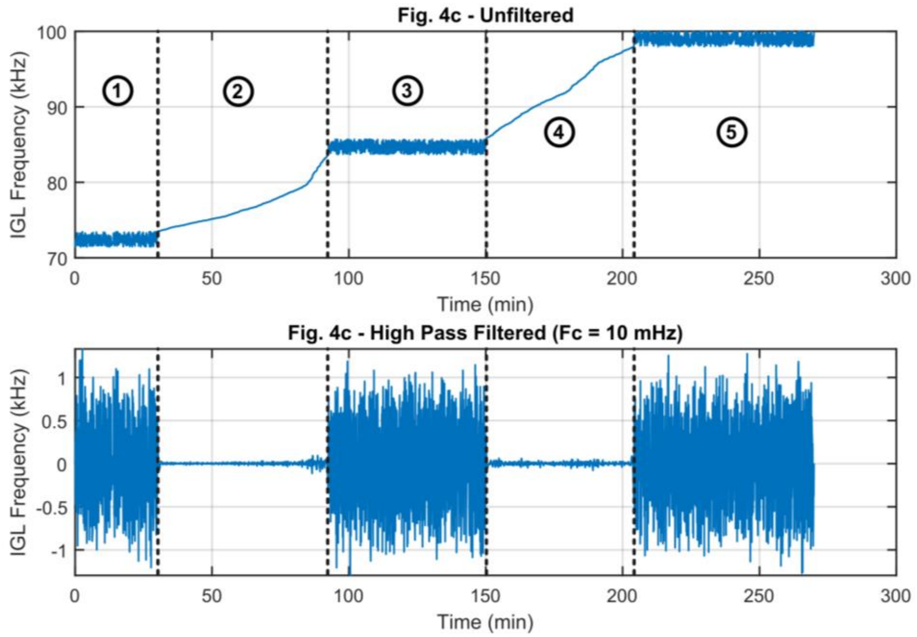

Supplementary Fig. 13. High pass filtered data from Figure 4c from the manuscript. (top) Data unfiltered. Domains 1, 3, and 5 represent the plateau sections. Domains 2 and 4 represent the raising step. (bottom) High pass filtered (Fc = 10 mHz)

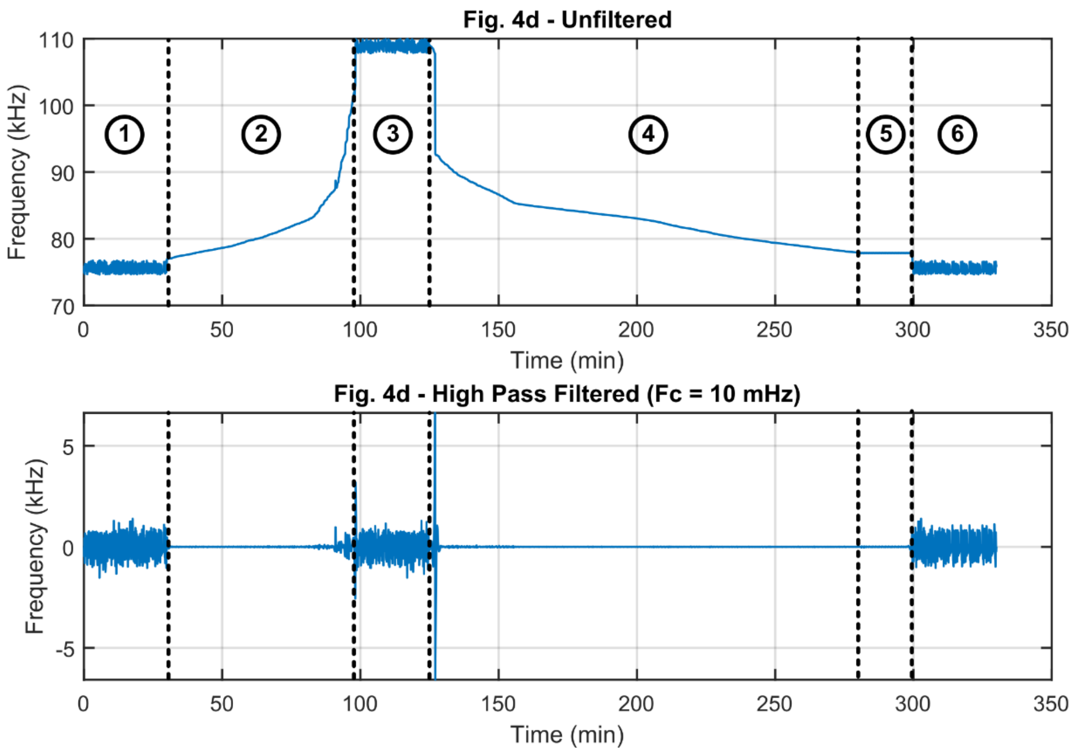

Supplementary Fig. 14. High pass filtered data from Figure 4d from the manuscript. (top) Data unfiltered. Domains 1 and 3 and 6 represent the plateau sections. Domain 2 represents the raising step. Domains 4 and 5 represent the decay step. (bottom) High pass filtered (Fc = 10 mHz).

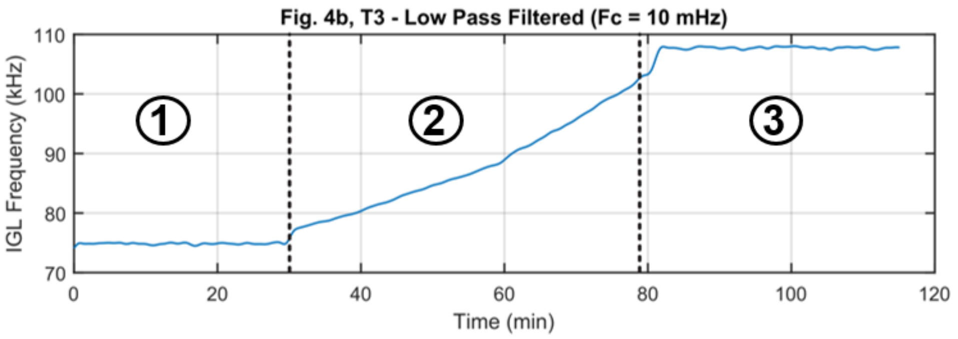

Supplementary Fig. 15. Low pass filtered data from Figure 4b-iii from the manuscript. (top) Data unfiltered. Domains 1 and 3 represent the plateau sections. Domain 2 represents the raising step. (middle) High pass filtered. (bottom) Low pass filtered data (Fc = 10 mHz).

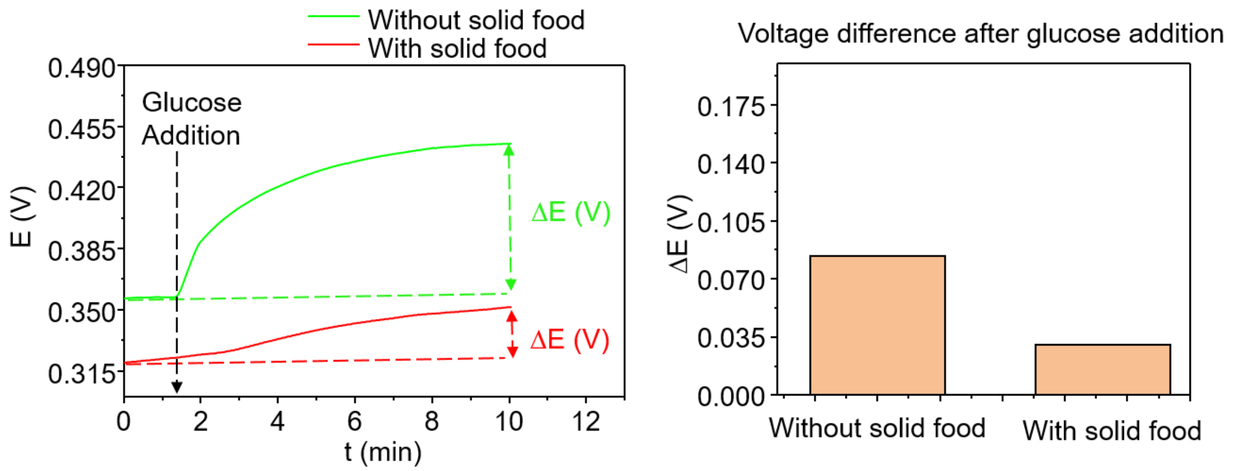

Supplementary Fig. 16. Solid food effect on the BFC sensor. (Left) Voltage signal recording was performed on the BFC sensor without solid food content (green curve) and with solid food (cracker crust) content (red curve). The voltage difference (ΔE) after glucose addition was calculated by the difference between the signal at 100 s and 600 s of the test. (Right). Voltage differences calculated after glucose addition without solid food and with solid food.
