# Supplementary material for: A self-powered ingestible wireless biosensing system for real-time in situ monitoring of gastrointestinal tract metabolites
Source: Nat Commun. 2022 Dec 1;13:7405. doi: 10.1038/s41467-022-35074-y (PMC9715945; doi:10.1038/s41467-022-35074-y)
Supplement: Supplementary file 2 — Reporting Summary [file 41467_2022_35074_MOESM2_ESM.pdf]

nature portfolio

Corresponding author(s): Patrick Mercier and Joseph Wang

Last updated by author(s): 10/07/2022

Reporting Summary

Nature Portfolio wishes to improve the reproducibility of the work that we publish. This form provides structure for consistency and transparency in reporting. For further information on Nature Portfolio policies, see our Editorial Policies and the Editorial Policy Checklist.

Statistics

For all statistical analyses, confirm that the following items are present in the figure legend, table legend, main text, or Methods section.

n/a	Confirmed	
☐	☒	The exact sample size (*n*) for each experimental group/condition, given as a discrete number and unit of measurement
☐	☒	A statement on whether measurements were taken from distinct samples or whether the same sample was measured repeatedly
☒	☐	The statistical test(s) used AND whether they are one- or two-sided<br>*Only common tests should be described solely by name; describe more complex techniques in the Methods section.*
☐	☒	A description of all covariates tested
☐	☒	A description of any assumptions or corrections, such as tests of normality and adjustment for multiple comparisons
☐	☒	A full description of the statistical parameters including central tendency (e.g. means) or other basic estimates (e.g. regression coefficient) AND variation (e.g. standard deviation) or associated estimates of uncertainty (e.g. confidence intervals)
☒	☐	For null hypothesis testing, the test statistic (e.g. *F*, *t*, *r*) with confidence intervals, effect sizes, degrees of freedom and *P* value noted<br>*Give P values as exact values whenever suitable.*
☒	☐	For Bayesian analysis, information on the choice of priors and Markov chain Monte Carlo settings
☒	☐	For hierarchical and complex designs, identification of the appropriate level for tests and full reporting of outcomes
☒	☐	Estimates of effect sizes (e.g. Cohen's *d*, Pearson's *r*), indicating how they were calculated

Our web collection on statistics for biologists contains articles on many of the points above.

Software and code

Policy information about availability of computer code

Data collection	For frequency data collection, by implementing a voltage-controlled oscillator (VCO), the integrated circuit can effectively convert the voltage from the biofuel cell corresponding to respective glucose concentration to frequency data. This frequency data is wirelessly transmitted using on-off keying (OOK) modulation, an on-chip power oscillator, and an off-chip 4-turn PCB antenna through mHBC. The mHBC focuses on the magnetic resonant coupling that leverages the body's low magnetic field losses and effectively turns the body into a dielectric waveguide or magnetic bubble. This is ideal for low-power on-body sensor networks interfacing with other on-body smart electronics. The data is then received by a receiver system connected to a laptop computer equipped with a oscilloscope (Keysight InfiniiVision MSOX 4024A) software for real-time data processing and visualization. A Metrohm Autolab potentiostat/galvanostat was used for data collection during in-vitro BFC potential studies.
Data analysis	For electrical characterization of the integrated circuit and studies using the integrated capsule device, a wireless mHBC receiver was used to down-convert the wireless received signal from the device to baseband. Next, the frequency data was extracted using an oscilloscope (Keysight InfiniiVision MSOX 4024A) software to analyze the data. To convert frequency data to glucose concentration, an internal calibration in-vitro calibration curve of the biofuel cell was used to obtained the intestinal glucose concentration.<br>For in-vitro biofuel cell potential studies, data analysis was performed using a Metrohm Autolab potentiostat/galvanostat and a Nova 2.0 software. The design of the 3D-printed capsule was made using SolidWorks® (x64 2016).

For manuscripts utilizing custom algorithms or software that are central to the research but not yet described in published literature, software must be made available to editors and reviewers. We strongly encourage code deposition in a community repository (e.g. GitHub). See the Nature Portfolio guidelines for submitting code & software for further information.

Data

Policy information about availability of data

All manuscripts must include a data availability statement. This statement should provide the following information, where applicable:

- Accession codes, unique identifiers, or web links for publicly available datasets
- A description of any restrictions on data availability
- For clinical datasets or third party data, please ensure that the statement adheres to our policy

The data of this study are available from the authors on reasonable request.

Human research participants

Policy information about studies involving human research participants and Sex and Gender in Research.

Reporting on sex and gender	We did not performed experiments on human participants
Population characteristics	n/a
Recruitment	n/a
Ethics oversight	n/a

Note that full information on the approval of the study protocol must also be provided in the manuscript.

Field-specific reporting

Please select the one below that is the best fit for your research. If you are not sure, read the appropriate sections before making your selection.

[x] Life sciences [ ] Behavioural & social sciences [ ] Ecological, evolutionary & environmental sciences

For a reference copy of the document with all sections, see nature.com/documents/nr-reporting-summary-flat.pdf

Life sciences study design

All studies must disclose on these points even when the disclosure is negative.

Sample size	Five farm pigs were used during the complete study. We do not perform any statistical analysis during the animal trials, therefore we do not predetermined the sample size.
Data exclusions	No data was excluded.
Replication	Data acquisition was carried out on multiple animals to verify the accuracy of the device. The experiments were performed every 2-3 weeks. Our replication studies were successful.
Randomization	The animals were selected based only in the range of age and weight. We do not perform any statistical analysis during the animal trials therefore, covariates were not relevant during the study.
Blinding	We do not perform any statistical analysis during the animal trials therefore, no blinding experiments were carried out. The data was directly processed after its collection.

Reporting for specific materials, systems and methods

We require information from authors about some types of materials, experimental systems and methods used in many studies. Here, indicate whether each material, system or method listed is relevant to your study. If you are not sure if a list item applies to your research, read the appropriate section before selecting a response.

Materials & experimental systems

n/a	Involved in the study
x	Antibodies
x	Eukaryotic cell lines
x	Palaeontology and archaeology
	x Animals and other organisms
x	Clinical data
x	Dual use research of concern

Methods

n/a	Involved in the study
x	ChIP-seq
x	Flow cytometry
x	MRI-based neuroimaging

Animals and other research organisms

Policy information about studies involving animals; ARRIVE guidelines recommended for reporting animal research, and Sex and Gender in Research

Laboratory animals	Farm pigs between three to eight months and weighing between 42 and 50 kg were selected for the study.
Wild animals	The study did not involve wild animals.
Reporting on sex	Sex of the animal was not considered for the experiments
Field-collected samples	The study did not involve field collected samples.
Ethics oversight	The experiments were conducted following the protocols approved by the Institutional Animal Care and Use Committee (IACUC) Office at the University of California, San Diego.

Note that full information on the approval of the study protocol must also be provided in the manuscript.
